# GLP-1 RAs for Treating Metabolic Dysfunction-Associated Steatosis Liver Disease: From GLP-1 Discovery to FDA Approval: A Comprehensive Narrative Review

**DOI:** 10.3390/ph19030408

**Published:** 2026-03-01

**Authors:** Olfa Khalifa, Abdelilah Arredouani

**Affiliations:** 1 Diabetes Research Center, Qatar Biomedical Research Institute, Hamad Bin Khalifa University, Qatar Foundation, Doha P.O. Box 34110, Qatar; 2 College of Health and Life Sciences, Hamad Bin Khalifa University, Qatar Foundation, Doha P.O. Box 34110, Qatar

**Keywords:** GLP-1, GLP-1R, GLP-1RA, MASLD

## Abstract

**Background:** Metabolic dysfunction-associated steatosis liver disease (MASLD), formerly termed nonalcoholic fatty liver disease (NAFLD), has emerged as the most prevalent cause of chronic liver disease worldwide. For decades, the absence of approved pharmacological therapies has hindered effective clinical management, leaving lifestyle modification and sustained weight reduction as the only recommended interventions. Yet, achieving and maintaining clinically meaningful weight loss remains an enduring challenge for most patients. Glucagon-like peptide-1 receptor agonists (GLP-1RAs), originally established as cornerstone therapies for type 2 diabetes (T2D) and obesity, have recently gained FDA approval for MASLD. Extensive mechanistic, preclinical, and clinical evidence demonstrates their ability to reduce hepatic steatosis, attenuate inflammatory pathways, and impede disease progression, establishing GLP-1RAs as the first pharmacological class with robust, multi-level efficacy in MASLD. **Scope of review:** This review synthesizes the evidence-based knowledge that led to the approval of GLP-1RAs for MASLD management. Integrating findings from (A) in vitro hepatocellular and multicellular models, (B) established animal models of steatosis-induced liver injury, and (C) clinical trials in patients with MASLD and its inflammatory subtype. We also discuss current limitations, unresolved questions, and future research priorities to optimize the therapeutic potential of GLP-1RAs in MASLD.

## 1. Introduction

Metabolic dysfunction-associated steatotic liver disease (MASLD) refers to a spectrum of liver conditions characterized by the accumulation of fat in liver cells, primarily due to underlying metabolic dysfunction [1,2]. MASLD can progress to more severe liver pathologies, including metabolic dysfunction-associated steatohepatitis (MASH), advanced fibrosis, cirrhosis, and hepatocellular carcinoma (HCC). The prevalence of MASLD is paralleling the rise in obesity and metabolic syndrome, affects approximately 25–30% of the global adult population, with a subset progressing to MASH or advanced liver fibrosis, highlighting the clinical and public health significance of this condition [3,4,5]. It is associated with central visceral adiposity except for a small proportion of lean patients [6,7], in whom genetic predisposition might play a crucial role in liver steatosis and fibrosis, insulin resistance (IR), and metabolic syndrome (MS). MASLD affects a significant proportion of the global population, with prevalence estimates ranging widely across regions, underscoring the challenges of lifestyle interventions and the potential need for pharmacological therapies such as GLP-1RAs. MASLD presents with heterogeneous patient profiles, ranging from lean to obese individuals, underscoring the need for therapeutic strategies that can effectively address this diversity.

Overall, up to 6.5% of MASLD patients meet the criteria of MASH [8], and the prevalence can be even higher in individuals with obesity, IR [9], or T2D, reaching up to 70–90% in these high-risk groups [8,9,10,11,12]. Advanced liver failure and HCC due to MASLD have positioned it as the second main cause of liver transplantation globally [13]. It is expected to become the primary cause of the obesity and T2D epidemics, which keep rising [14,15]. Addressing MASLD is paramount because of its close link with metabolic comorbidities such as obesity, IR, and T2D, chronic kidney disease, polycystic ovary syndrome, dyslipidemia, and cardiovascular disease, the leading cause of death in this population. Given the difficulty of achieving sustained weight loss and lifestyle modifications in many patients, there is a clear need for pharmacological interventions, such as GLP-1RAs, to manage MASLD effectively. MASLD patients present with heterogeneous patient profiles, ranging from lean to obese individuals, underscoring the need for therapeutic strategies that can effectively address this diversity.

The multisystemic nature of MASLD necessitates an urgent, multidisciplinary approach to screening, prevention, and management, as failure to address this condition may significantly burden healthcare systems and compromise long-term patient outcomes [6]. As of 15 August 2025, Resmetirom remained the sole pharmacological agent approved by the FDA for the treatment of MASH with liver fibrosis [16]. On 15 August, the FDA approved semaglutide (Wegovy) (a GLP-1RA) injection for the treatment of adults with MASH and moderate-to-advanced hepatic fibrosis, characterized by excessive deposition of scar tissue in the liver (FDA, 2025). Wegovy was previously approved for obesity or overweight and to reduce cardiovascular events, such as heart attacks, in individuals at high risk of these events. The recent FDA approval of semaglutide for the treatment of MASH represents the culmination of an extensive body of evidence derived from in vitro studies, preclinical animal models, and multiple clinical trials. Additionally, this review integrates mechanistic insights from cellular and animal studies with human clinical data to provide a comprehensive perspective. We also highlight current knowledge gaps and future research directions, offering guidance not addressed in previous reviews.

Herein, we review the spectrum of evidence encompassing these various investigations that ultimately led to the FDA approval of semaglutide for the treatment of MASH with moderate to advanced fibrosis [17,18,19,20,21,22,23,24].

## 2. GLP-1 and Its Receptor

Glucagon-like peptide-1 (GLP-1) is a 30-amino acid incretin hormone derived from the differential processing of proglucagon in enteroendocrine L-cells of the distal small intestine and colon [25]. Nutrient ingestion, particularly of carbohydrates and fats, rapidly increases plasma GLP-1 levels. However, its short half-life of 1–2 min, due to enzymatic degradation by dipeptidyl peptidase-4 (DPP4), limits systemic bioavailability to ~10–15%. Despite this, GLP-1 exerts potent physiological effects, including stimulation of glucose-dependent insulin secretion, inhibition of glucagon release, delayed gastric emptying, and promotion of satiety, thereby integrating glucose homeostasis with energy balance [26,27]. Beyond its role in metabolic regulation, GLP-1 also exhibits anti-inflammatory and cytoprotective effects on various tissues [28,29,30,31]. The actions of GLP-1 are mediated through the GLP-1 receptor (GLP-1R), a class B G protein-coupled receptor consisting of an extracellular N-terminal domain for ligand binding, seven transmembrane helices, and an intracellular C-terminal domain responsible for signal transduction [32]. GLP-1R is abundantly expressed in pancreatic β-cells, where it enhances insulin biosynthesis, secretion, and survival. Still, it is also widely distributed in the brain, kidney, heart, gastrointestinal tract, endothelial cells, neurons, astrocytes, and immune cells, underscoring its pleiotropic functions [30,33,34,35]. ’The presence of GLP-1 receptors in human hepatocytes remains debated, with some studies reporting their detection in human and animal models [36,37], while others do not; clarifying their expression is central to determining whether GLP-1RAs act directly on the liver or indirectly through extrahepatic pathways. Such inconsistencies may stem from methodological variability, including antibody specificity, tissue processing, and detection sensitivity. Until robust evidence is available, conclusions regarding their mechanisms in liver metabolism should be interpreted with caution. Emerging approaches, including single-cell RNA sequencing and refined in situ hybridization, may provide the necessary resolution to settle this controversy [38]. Overall, current evidence suggests that while GLP-1 receptors are present in hepatocytes at low levels, their activation may indirectly modulate hepatic metabolism and inflammation, highlighting a potential therapeutic role in MASLD.

## 3. Discovery and Development of GLP-1R Agonists

The clinical use of native GLP-1 is limited by its 1–2 min half-life, which necessitates continuous infusion. In the early 1990s, investigators identified Exendin-4, the first GLP-1 receptor agonist (GLP-1RA), from the saliva of the Gila monster *Heloderma suspectum* [39,40]. Despite only ~53% sequence similarity to human GLP-1, Exendin-4 is highly resistant to DPP-4 degradation, extending its half-life to ~2.5 h [41] and enabling practical twice-daily dosing. Its synthetic form, Exenatide (marketed as Byetta), received FDA approval in 2005, initiating a new era of incretin-based therapy for T2D.

Over the years, subsequent efforts produced multiple long-acting GLP-1RAs. Liraglutide (Victoza, approved 2010; Saxenda, approved 2014) utilizes a fatty-acid side chain to promote albumin binding, resulting in a half-life of ~13 h, which is suitable for once-daily dosing. Exenatide extended-release (Ex-ER), approved in 2012, further prolongs exposure to approximately 168 h, allowing for once-weekly (QW) administration [42]. Dulaglutide (Trulicity), approved in 2014, achieves a ~5-day half-life through fusion with an engineered IgG4 Fc fragment, also enabling QW dosing [43].

Semaglutide (Ozempic, approved 2017; Rybelsus, approved 2019; Wegovy, approved 2021) set new efficacy standards for the class. Structural modifications that enhance similarity to native GLP-1 while protecting against DPP-4 yield a half-life of ~1 week and superior glycemic, weight-loss, and cardiovascular benefits [44]. These agents consistently lower HbA1c, induce clinically meaningful weight loss, and, particularly, semaglutide reduces major cardiovascular events [44].

A major advance occurred with the 2022 approval of Tirzepatide (Mounjaro), the first dual GIP/GLP-1 receptor agonist. With a half-life of ~5 days, it delivers synergistic metabolic effects and has demonstrated unprecedented improvements in both glycemic control and weight reduction [45,46,47]. Emerging agents, including incretin-based therapies such as Retatrutide (GIP/glucagon/GLP-1) [48] and NA-931 (IGF-1/GLP-1/GIP/glucagon) [49], show promise for MASLD treatment and are currently in mid- to late-stage clinical development; however, they remain investigational and are not yet approved for clinical use.

Modern incretin drugs now offer broad metabolic benefits extending beyond glycemic control to weight reduction and cardiovascular protection. This evolution has shifted care toward a proactive, disease-modifying strategy with significant long-term impact. Ongoing research is expanding their potential applications to MASLD, neurodegenerative diseases, and alcohol and drug addictions [42].

## 4. Effects of GLP-1RA on Liver Fat Content

As mentioned above, the expression of GLP-1R in hepatocytes is still debated. Several studies report the absence of detectable functional GLP-1R in human or rodent hepatocytes, suggesting that direct hepatic actions are unlikely [37]. This supports the view that improvements in liver fat are primarily indirect, driven by weight loss, enhanced insulin sensitivity, and reduced adipose-derived lipid flux. Other reports suggest low or context-dependent receptor expression, leaving the possibility of limited direct signaling open. The following sections will summarize key findings from cell, animal, and human studies evaluating the impact of GLP-1RAs on liver fat content.

### 4.1. Cell Models

Significant advancements in understanding the effect of GLP-1RAs on hepatic lipid accumulation were made through various in vitro cell models. These studies provide a foundation for understanding the mechanistic action of these drugs at the cellular level.

#### 4.1.1. Studies on Hepatocytes

In vitro cell models provide a controlled platform for investigating how GLP-1RAs influence hepatic steatosis, utilizing primary hepatocytes and widely employed hepatic cell lines, such as HepG2, Huh7, L02, AML12, as well as co-cultures of Kupffer cells and JS-1. Across these systems, GLP-1RAs consistently reduce lipid accumulation and fatty acid (FFA) overload, effects that are likely mediated by systemic metabolic improvements rather than direct hepatic actions [50]. Steatosis is typically induced using palmitic acid (PA), which triggers lipotoxicity, ER stress, and inflammation [51] or oleic acid (OA), which promotes lipid deposition with comparatively lower inflammatory signaling [52,53,54]. A common set of mechanistic outcomes emerges from these studies: GLP-1RAs reduce intracellular lipid accumulation, enhance fatty acid oxidation, suppress lipogenic gene expression, mitigate oxidative and ER stress, improve insulin sensitivity, and diminish inflammatory responses. In PA-induced HepG2 steatosis models, liraglutide (100 nM–1 μM, 24 h) reduced lipid droplet content by 30–50% [55]. This effect was accompanied by a 40–60% suppression of SREBP-1c and FAS, coupled with increased expression of PPARα, the principal regulator of β-oxidation [56,57]. Liraglutide also attenuated PA-driven oxidative stress, lowering ROS by ~35% and activating Nrf2-dependent antioxidant genes such as HO-1 and NQO1 [58,59]. Furthermore, it reduced ER stress markers, notably CHOP, ATF4, and GRP78 [60].

Exendin-4 has shown similar lipid-lowering activity. In HepG2 cells exposed to 400 μM PA for 24 h, Ex-4 (100 nM) markedly suppressed PA-induced upregulation of SREBP-1c, PPARγ, SCD1, FAS, ACC, DGAT1, and DGAT2. Mechanistic analyses revealed activation of the Wnt/β-catenin pathway, with increased GSK-3β phosphorylation and nuclear β-catenin/TCF4 accumulation; conversely, β-catenin silencing or pharmacologic inhibition eliminated Ex-4’s effects [52]. In OA-induced steatosis, Ex-4 (200 nM, 3 h) significantly reduced lipid accumulation and downregulated FABP1 and its transcriptional activator FOXA1. β-catenin knockdown abolished these effects and further enhanced OA-driven SREBP-1 and TCF4 induction [53].

Taken together, these in vitro findings demonstrate that GLP-1RAs directly ameliorate hepatic steatosis through the coordinated modulation of lipid metabolic and stress response pathways. They suppress de novo lipogenesis, limit fatty acid uptake and transport, alleviate oxidative and ER stress, and promote fatty acid catabolism [61]. Table 1. summarizes the major steatosis-inducing agents, GLP-1RAs evaluated, study references, and key mechanistic outcomes.

#### 4.1.2. Mechanisms of Action at the Cellular Level

The hepatoprotective mechanisms of GLP-1RAs are largely defined through in vitro studies using human hepatocyte models, including HepG2, Huh7, and primary human hepatocytes (PHHs) [61].

GLP-1R activation triggers Gαs-dependent cAMP production and PKA activation [72]. One major consequence is the inhibition of hepatic gluconeogenesis: in palmitate-treated HepG2 cells and PHHs, liraglutide activated the cAMP/PKA pathway, inducing CREB phosphorylation and the nuclear receptor SHP, which represses FOXO1 and HNF4α, reducing PEPCK and G6Pase expression by ~50% [73,74].

GLP-1RAs also enhance insulin sensitivity. Exenatide restored PPARγ expression, reduced JNK activation, and increased phosphorylation of PKA, AMPK, and Akt, while enhancing PPARα activity, collectively promoting β-oxidation and improving hepatocyte insulin signaling [75]. Reduced JNK activity limits inhibitory serine phosphorylation of IRS proteins. Beyond glucoregulation, GLP-1RAs exert direct anti-inflammatory actions. In TNF-α–stimulated Huh7 cells, semaglutide (0.1 µM) prevented IκBα phosphorylation and NF-κB p65 nuclear translocation, decreasing IL-6 and TNF-α secretion by ~60% [76,77,78]. Liraglutide similarly attenuated ER stress–induced JNK/NF-κB activation in PHHs by downregulating GRP78 and CHOP [79]. In OA-induced steatotic HepG2 cells, liraglutide activated AMPK (Thr172), leading to ACC inhibition, reduced malonyl-CoA, CPT1A activation, and enhanced β-oxidation [80]. AMPK also blocked nuclear SREBP-1c translocation, resulting in a ~50% reduction in FASN and SCD1 expression [81,82].

**Figure 1:**
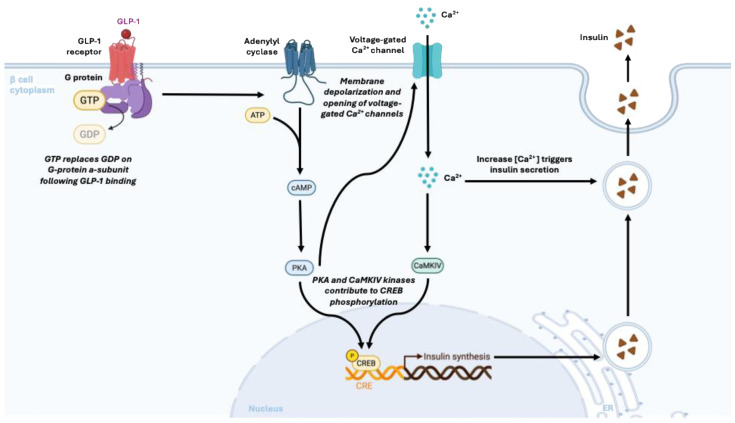
Molecular Mechanism of Action of Glucagon-like Peptide-1 (GLP-1) in Pancreatic β-Cells. GLP-1 binds to its receptor on the β-cell membrane, activating the G protein-coupled signaling pathway by replacing GDP with GTP on the α-subunit. This activates adenylate cyclase, leading to the production of cAMP, which in turn activates PKA. The signaling cascade causes membrane depolarization and opening of voltage-gated Ca^2+^ channels, resulting in Ca^2+^ influx that triggers insulin secretion. Concurrently, Ca^2+^ activates CaMKIV, and together with PKA, phosphorylates CREB in the nucleus, enhancing insulin gene transcription. This integrated mechanism promotes both rapid insulin release and sustained insulin synthesis, supporting glucose homeostasis.

In summary, GLP-1RAs act directly on hepatocytes through: (a) cAMP/PKA/CREB/SHP-mediated suppression of gluconeogenesis, by the activation in hepatocytes and other liver cells engages key signaling pathways-including AMPK, PKA, and NF-κB- that collectively modulate lipid metabolism, inflammation, and cellular stress responses, providing a mechanistic basis for their therapeutic effects in MASLD; (b) enhancement of IRS-2/Akt insulin signaling with reduced JNK activity; (c) AMPK-driven inhibition of lipogenesis and stimulation of β-oxidation; and (d) inhibition of NF-κB and JNK pathways to reduce inflammation. These coordinated pathways support the therapeutic benefit of GLP-1RAs in MASLD and MASH. Figure 1. Shows the molecular mechanism of action of GLP-1.

### 4.2. Animal Models

#### 4.2.1. Rodent Studies

A wide range of rodent models has been developed to study MASLD, which can be broadly categorized into genetic, dietary, and chemical approaches. Genetic models, such as ob/ob, db/db, ApoE^−/−^, Ldlr^−/−^, foz/foz, and LIR^−/−^ mice, help dissect inherited mechanisms linked to obesity, insulin resistance, and disordered lipid metabolism. Dietary models, including methionine- and choline-deficient (MCD) diets, high-fat diets, and sugar- or cholesterol-enriched combinations, capture the nutritional triggers that drive human disease and are widely used [83]. Chemical models employing streptozotocin, CCl_4_, or DEN, alone or paired with diets, accelerate progression toward inflammation, fibrosis, or cancer. Collectively, these complementary systems replicate different stages of MASLD, providing platforms for studying mechanisms and evaluating interventions. Mice and rats remain the primary species used, and each model offers distinct mechanistic insight [84]. Rodent systems have also been central for examining GLP-1 activity: in diet-induced obese, ob/ob, and MCD models [85], multiple GLP-1RAs (liraglutide, semaglutide, exenatide) consistently improve steatosis and inflammation [86]. Finally, Tirzepatide, a once-weekly injectable dual GIP/GLP-1 receptor agonist originally developed for type 2 diabetes and obesity, has shown promise in preclinical MAFLD models by reducing hepatic triglycerides, lowering liver enzymes, and modulating fatty acid metabolism in high-fat diet mice, suggesting hepatoprotective mechanisms beyond weight loss [87].

#### 4.2.2. Zebrafish Models

In recent years, zebrafish have emerged as a novel vertebrate model for MASLD [88]. Zebrafish share high genetic homology with humans and exhibit conserved lipid metabolic pathways, while offering unique experimental advantages such as rapid development, optical transparency, and suitability for high-throughput chemical screening [89]. MASLD in zebrafish can be induced by dietary interventions (high-fat or cholesterol-rich feeding), exposure to hepatotoxic chemicals, or targeted genetic manipulations affecting lipid metabolism [88,90]. These models have been successfully used to study lipid droplet dynamics, hepatic oxidative stress, and the interaction between liver and gut microbiota. Moreover, zebrafish are highly amenable to live imaging, enabling dynamic visualization of lipid accumulation and inflammatory responses in real time [91]. While GLP-1RA studies in zebrafish are still limited, preliminary data suggest that these agents improve hepatic lipid metabolism and reduce inflammatory signaling. Given their scalability, zebrafish models are particularly promising for preclinical drug discovery pipelines [92].

#### 4.2.3. Large Animal Models

While less extensively studied than rodent models, large animal studies offer important translational insights into the effects of GLP-1RA on hepatic metabolism. In Ossabaw swine, which is a breed of pig derived from a population of feral pigs on Ossabaw Island in the USA, rendered obese and IR through high-fat, high-fructose feeding, extended-release exenatide treatment (2 mg/kg/week for 16 weeks) significantly improved hepatic insulin sensitivity and reduced intrahepatic lipid content by approximately 35%, as quantified by magnetic resonance spectroscopy [93]. Similarly, studies in non-human primates with spontaneous obesity and IR have demonstrated that liraglutide treatment (0.1 mg/kg/day for 12 weeks) reduces hepatic steatosis and decreases markers of oxidative stress and inflammation [94].

These large animal models are particularly valuable for confirming the relevance of mechanisms identified in rodent studies, including enhanced hepatic insulin signaling through IRS-2/Akt pathways and reduced activation of pro-inflammatory JNK/NF-κB signaling cascades. The concordance of findings across rodent and large animal models strengthens the evidence for GLP-1RA-mediated hepatoprotection, supporting the translation of these effects to human physiology [95].

Zebrafish and large animal models provide complementary insights into rodent studies, offering advantages in modeling complex metabolic and liver processes relevant to humans. Their use helps bridge preclinical findings with potential clinical outcomes, enhancing the translational relevance of MASLD research.

#### 4.2.4. Effects on Hepatic Steatosis and Inflammation in Animal Models

Rodent studies consistently demonstrate that GLP-1RAs enhance hepatic lipid metabolism primarily through AMPK activation, which phosphorylates and inhibits ACC, thereby promoting fatty acid oxidation and suppressing lipogenesis [96]. In DIO mice, liraglutide downregulated SREBP-1c and its lipogenic targets, reducing de novo lipogenesis and hepatic triglycerides [97]. GLP-1RAs also enhance IRS-2/Akt signaling, increasing glycogen synthesis and suppressing gluconeogenic genes (PEPCK, G6Pase), while reducing JNK activity to restore hepatic insulin sensitivity [25].

GLP-1RAs exert strong anti-inflammatory effects: liraglutide lowers hepatic TNF-α, IL-1β, MCP-1, and macrophage infiltration [98], while exenatide suppresses NF-κB signaling and reduces cytokine transcription. By alleviating ER stress, GLP-1RAs limit DAMP release and activation of Kupffer cells [99]. They may also attenuate fibrogenesis; exenatide inhibits TGF-β/Smad signaling and reduces extracellular matrix deposition [100]. Beyond rodents, ossabaw swine treated with extended-release exenatide exhibited improved hepatic insulin sensitivity and ~35% lower liver fat, accompanied by enhanced mitochondrial function [101]. In non-human primates, liraglutide reduced steatosis, oxidative stress, and inflammatory markers [102]. These cross-species findings reinforce the translational relevance of GLP-1RA-mediated hepatoprotection. Table 2 summarizes key in vivo studies conducted over the past decade across mice, rats, zebrafish, and other animal models.

### 4.3. GLP-1RA Therapy for MASLD: Clinical Trials Overview

Given the strong metabolic underpinnings of MASLD, increasing attention has focused on therapies that target systemic metabolic dysfunction. Among these, GLP-1RAs have gained prominence as a potential therapy for MASLD due to their pleiotropic metabolic effects, including appetite suppression, delayed gastric emptying, improved insulin sensitivity, reduced inflammation and oxidative stress, and favorable effects on lipid metabolism, all of which are mechanistically relevant to MASLD pathogenesis. Initial clinical trials examining the effects of GLP-1RAs on T2D and obesity demonstrated a reduction in hepatic fat content, suggesting that these agents could be repurposed for the treatment of MASLD. Consequently, dedicated clinical trials were subsequently conducted in patients with biopsy- or imaging-proven MASLD.

Early clinical evidence supporting the hepatic benefits of GLP-1RAs was provided by the LEAN (Liraglutide Efficacy and Action in NASH) trial, a landmark 48-week, double-blind, randomized, placebo-controlled study evaluating liraglutide in patients with biopsy-proven MASH. In this trial, 52 overweight individuals received once-daily subcutaneous liraglutide (1.8 mg) or placebo. The primary endpoint was histological resolution of MASH without worsening of fibrosis. Liraglutide treatment resulted in a significantly higher rate of MASH resolution compared with placebo (39% vs. 9%; *p* = 0.019), while also reducing the proportion of patients experiencing fibrosis progression (9% vs. 36%; *p* = 0.04) [119]. Importantly, these histological improvements occurred in the absence of excessive adverse effects, supporting both the efficacy and safety of liraglutide in this patient population. The LEAN trial provided the first proof-of-concept evidence that GLP-1RA therapy could favorably modify liver histology in NASH.

Subsequent trials further explored the effects of GLP-1RAs on hepatic fat content (HFC), a key driver of disease progression. The Lira-M study investigated the impact of liraglutide (1.2 mg daily) compared with insulin-based therapy on liver fat accumulation in 68 patients with inadequately controlled T2D. Hepatic fat was quantified using proton magnetic resonance spectroscopy (1H-MRS), a highly sensitive and non-invasive technique. After six months of treatment, patients receiving liraglutide experienced a marked 31% reduction in HFC (*p* < 0.0001), whereas no significant change was observed in the insulin-treated group [120]. Notably, the reduction in hepatic fat strongly correlated with weight loss, suggesting that the hepatic benefits of liraglutide are at least partially mediated by its effects on body weight and energy balance. However, additional mechanisms, including direct effects on hepatic lipid metabolism and insulin signaling, may also contribute.

The therapeutic potential of other GLP-1RAs has been evaluated in similar clinical contexts. The D-LIFT trial assessed the effects of once-weekly dulaglutide in patients with T2D and MASLD. This 24-week, open-label, randomized controlled trial enrolled 64 participants, who were assigned to receive either dulaglutide in addition to standard care or standard care alone. Dulaglutide treatment led to a significant absolute reduction in hepatic fat content of 3.5% and a relative reduction of 26.4%, which was more than double the reduction observed in the control group [121]. In addition to reducing liver fat, dulaglutide significantly lowered serum gamma-glutamyl transferase (GGT) levels, indicating an improvement in hepatocellular injury. These findings reinforced the concept that GLP-1RAs exert consistent beneficial effects on liver fat and biochemical markers of liver health across different agents within the drug class.

Further support for liraglutide’s hepatic benefits was provided by a randomized trial conducted by Guo and colleagues, which compared liraglutide with insulin glargine or placebo in patients with MASLD and poorly controlled T2D receiving metformin [122]. Over a 26-week treatment period, liraglutide significantly reduced hepatic fat content, whereas insulin glargine produced more modest and non-significant changes [122]. Additionally, liraglutide treatment was associated with significant reductions in alanine aminotransferase (ALT), aspartate aminotransferase (AST), and homeostatic model assessment of insulin resistance (HOMA-IR), underscoring its favorable impact on both hepatic inflammation and systemic insulin sensitivity.

Beyond individual trials, post hoc analyses of large clinical programs have further substantiated the hepatic effects of GLP-1RAs. An analysis of the AWARD trials evaluated liver enzyme changes in a large subgroup of patients with T2D and MASLD or suspected MASH treated with dulaglutide. Over a six-month period, dulaglutide significantly reduced ALT, AST, and GGT levels compared with the placebo, suggesting improved liver health at a population level [123]. Although liver histology was not assessed in this analysis, improvements in liver enzymes are widely considered surrogate markers of reduced hepatic inflammation and steatosis.

More recently, next-generation incretin-based therapies have expanded the therapeutic landscape. Efinopegdutide, a dual GLP-1/glucagon receptor co-agonist, has demonstrated particularly potent effects on hepatic fat reduction. In a 24-week randomized, active-controlled trial, efinopegdutide achieved a 72.7% relative reduction in hepatic fat content compared with 42.3% for semaglutide, as measured by MRI-based techniques [124]. Both agents also produced significant weight loss; however, the greater reduction in liver fat observed with efinopegdutide suggests that mechanisms beyond weight loss, such as enhanced hepatic lipid oxidation mediated by glucagon receptor activation, may also play a role.

Collectively, these clinical data demonstrate that GLP-1RAs consistently improve hepatic fat accumulation, liver enzyme profiles, and, in some cases, histological features of steatohepatitis and fibrosis. While weight loss appears to be a major driver of these benefits, emerging evidence suggests that GLP-1RAs may also exert direct hepatic effects, including modulation of de novo lipogenesis, enhancement of fatty acid oxidation, and attenuation of inflammatory signaling pathways. These multifaceted actions make GLP-1RAs desirable candidates for addressing the complex pathophysiology of MASLD.

Finally, the SYNERGY-NASH (NCT04166773) phase 2b randomized, double-blind, placebo-controlled trial enrolled 190 adults with biopsy-confirmed MASH and stage F2–F3 fibrosis, including participants with and without T2D, and tested subcutaneous tirzepatide at 5 mg, 10 mg, and 15 mg once weekly versus placebo for 52 weeks, with dosing escalated from 2.5 mg to target levels [125]. Tirzepatide produced robust reductions in body weight and significant improvements in liver biochemistries, as well as in noninvasive biomarkers of hepatic steatosis and fibrosis, compared with placebo [125]. Despite these encouraging mid-stage clinical results and ongoing regulatory evaluation, tirzepatide is not currently approved by the U.S. FDA for the treatment of MASH/MASLD. The clinical trials included in this review vary in study design, duration, and primary endpoints. This variability should be considered when interpreting the reported efficacy and safety outcomes of GLP-1 receptor agonists in MASLD.

In summary, a growing body of clinical evidence supports the use of GLP-1RAs as therapeutic agents for MASLD. These drugs consistently reduce hepatic fat content, improve biochemical markers of liver injury, and, in selected studies, induce histological resolution of steatohepatitis. Their favorable safety profile, combined with established benefits in weight management, glycemic control, and cardiovascular risk reduction, positions GLP-1RAs as strong candidates to fill the current therapeutic gap in MASLD. Nevertheless, further large-scale, long-term trials with histological endpoints are needed to define optimal dosing strategies, identify patient subgroups most likely to benefit, and determine the durability of treatment responses. Continued investigation into their extra-hepatic effects, particularly in the brain, may further broaden the clinical utility of this versatile drug class. Table 3 summarizes key clinical trials that evaluated GLP-1RAs in patients with MASLD/NASH, highlighting study design, treatment regimens, and primary hepatic outcomes.

#### 4.3.1. Challenges and Future Direction

The recent FDA approval of semaglutide for the treatment of MASH with fibrosis represents a significant milestone in the management of metabolic liver disease. Semaglutide has received FDA approval in multiple formulations for distinct indications: Ozempic (2017) for type 2 diabetes, Rybelsus (2019) as an oral GLP-1RA for diabetes, and Wegovy (2021) for chronic weight management. The recent regulatory approval of Wegovy for adults with MASH and moderate-to-advanced hepatic fibrosis reflects the growing body of preclinical, mechanistic, and clinical evidence supporting the therapeutic potential of GLP-1RAs in MASLD/MASH, while several other GLP-1RAs and emerging multi-agonist therapies remain under regulatory evaluation for this indication. Mechanistically, GLP-1RAs exert multiple beneficial effects, including enhancing insulin sensitivity, reducing hepatic lipotoxicity, modulating inflammatory and fibrotic signaling pathways, promoting weight loss, and improving lipid and glucose metabolism. Key clinical trials have demonstrated that semaglutide and other GLP-1RAs can induce significant resolution of MASH without worsening fibrosis, accompanied by substantial reductions in body weight, liver enzymes, and non-invasive imaging biomarkers of steatosis and fibrosis. These trials have included heterogeneous populations with obesity, T2D, and varying stages of fibrosis, providing robust evidence of efficacy across multiple metabolic subgroups. Despite these advances, several challenges remain. Patient heterogeneity in MASLD and MASH complicates treatment response, as genetic background (e.g., PNPLA3 and TM6SF2 variants), comorbid metabolic disease, age, sex, and fibrosis stage may influence therapeutic outcomes. In addition, long-term data on clinically meaningful endpoints, including cirrhosis progression, hepatic decompensation, liver-related mortality, and cardiovascular outcomes, remain limited, which constrains definitive assessment of disease-modifying benefits.

Gastrointestinal side effects, including nausea, vomiting, and diarrhea, may limit adherence, particularly in patients requiring high GLP-1RA doses for optimal efficacy. The high cost of GLP-1RAs also restricts access globally, and the lack of validated non-invasive biomarkers (e.g., MRI-based liver fat quantification, PNPLA3 genetic variants, serum metabolite panels) makes it challenging to tailor therapy to individual disease activity or predict long-term response. Future directions emphasize the evaluation of GLP-1RAs to diverse MASLD populations, including the understudied lean MASLD subgroup, which often exhibits non-obesity-related disease drivers such as genetic variants, sarcopenia, or metabolic dysfunction. In lean MASLD, GLP-1RAs may exert distinct effects due to differences in body composition, insulin sensitivity, and metabolic profiles compared to obese patients, guiding optimized therapeutic strategies.

Dedicated trials are needed where weight-loss–mediated mechanisms may be less effective. Investigating combination therapies targeting multiple pathogenic pathways, including fibrogenesis, lipid metabolism, or oxidative stress, may enhance outcomes. Development of oral or multi-agonist formulations could improve adherence, tolerability, and efficacy. Precision medicine approaches using genetic, metabolomic, and microbiome profiling can enable individualized treatment strategies. Generating real-world evidence on long-term safety, effectiveness, and cost–benefit profiles will inform policy and clinical practice. Finally, combination therapies with SGLT2 inhibitors or FXR agonists may provide synergistic metabolic and hepatic benefits, improving overall MASLD treatment efficacy. Future studies may explore combination therapies, such as GLP-1 receptor agonists with SGLT2 inhibitors or FXR agonists, to enhance metabolic and hepatic outcomes. These approaches could provide synergistic benefits and improve overall treatment efficacy in MASLD.

#### 4.3.2. General Conclusions

The comprehensive body of in vitro, in vivo, and clinical evidence reviewed herein, spanning preclinical models to pivotal human trials, supports the potential of GLP-1RAs in managing MASLD and MASH. Preclinical studies consistently demonstrate that GLP-1RAs reduce hepatic lipid accumulation, attenuate inflammatory pathways, and modulate profibrotic signaling, providing mechanistic insight into their hepatoprotective effects. These findings are further supported by randomized clinical trials enrolling diverse cohorts of patients with obesity, type 2 diabetes, and varying stages of fibrosis, in which GLP-1RAs were associated with improvements in histological features of MASH, reductions in body weight, liver enzymes, and noninvasive markers of hepatic fat and fibrosis.

These data contributed to the FDA’s 2025 approval of semaglutide (Wegovy) for MASH with moderate to advanced fibrosis, representing an important step in the development of pharmacological therapies for MASLD. While this approval is promising, these benefits remain potential and require further validation, as long-term effects on clinically meaningful endpoints such as cirrhosis progression, liver-related mortality, and cardiovascular outcomes are not yet fully established.

Despite these advances, challenges remain. Common gastrointestinal side effects, including nausea and diarrhea, may affect adherence but can often be mitigated through gradual dose escalation. Long-term safety data are limited, and high costs and restricted access may impede widespread use. The efficacy of GLP-1RAs in heterogeneous patient populations, particularly lean MASLD patients, requires further investigation. Future directions include combination therapies targeting multiple pathogenic mechanisms, the identification of precision biomarkers to guide individualized therapy, and expansion of clinical trials across diverse MASLD subgroups. Addressing these areas is essential to fully realize the therapeutic potential of GLP-1RAs across the spectrum of metabolic liver disease.

Finally, GLP-1 RAs show promise in improving metabolic and hepatic outcomes in MASLD and MASH; however, long-term studies and additional research are needed to fully establish their efficacy, safety, and impact on clinically meaningful endpoints.

## Figures and Tables

**Table 1:** Key in Vitro studies elucidating GLP-1RA mechanisms in Hepatic steatosis.

Cell Line	Steatosis Inducer	GLP1-RA Treatment	Majors Finding	Year	Citation
**Primary human hepatocytes**	PA OA EA	Exendin-4	- GLP-1 proteins appear to protect hepatocytes from fatty acid-related death by prohibition of a dysfunctional ER stress response; and reduce fatty acid accumulation, by activation of both macro-and chaperone-mediated autophagy. - These findings provide a novel role for GLP-1 proteins in halting the progression of more aggressive lesions from underlying steatosis in humans afflicted with MASLD.	2024 2022	[62,63,64,65]
**Primary mice Kupffer cells**	PA	Liraglutide	- Primary mouse Kupffer cells have been used to study the role of liver macrophages in MASLD. - Studies suggest that GLP-1RAs can modulate inflammatory responses in Kupffer cells, leading to reduced hepatic inflammation.	2023	[66]
**Huh7**	OA	GLP-1 Liraglutide	- Studies indicate that treatment with GLP-1RAs leads to reduced inflammation and lipid accumulation through liraglutide.	2022	[67]
**HepG2**	OA PA LPS	Exendin-4 AWRK6 Liraglutide	- Studies have shown that Exendin-4 can improve fatty acid oxidation - Research has demonstrated that GLP-1RAs can downregulate lipogenic gene expression and enhance insulin sensitivity in HepG2 cells. -Liraglutide promotes dose-dependent apoptosis of HepG2 cells, likely by activating the JNK signaling pathway.	2022 2021 2022	[53,67,68]
**L-02**	FFA mixture	Liraglutide	- Normal human liver cell line used to assess hepatocyte function and viability. - Liraglutide reduces lipid accumulation in steatotic L-02 cells by enhancing autophagy - GLP-1RAs have been shown to improve metabolic profiles and reduce oxidative stress in L-02 cells treated with fatty acids.	2014	[69]
**AML12**	PA	Liraglutide Semaglutide	- Murine hepatocyte cell line that mimics liver function and steatosis. - Research using AML12 has indicated that GLP-1RAs can enhance fatty acid oxidation and mitigate lipotoxicity.	2022 2024	[70,71]

EA: Elaidic Acid; LPS: Lipopolysaccharide; FFA: Free Fatty Acid; PA: Palmitic Acid; OA: Oleic Acid.

**Table 2:** Key In Vivo experimental models for evaluating GLP-1RA treatment in MASLD/MASH.

Animal Model	Steatosis Inducer	GLP-1RA	Major Findings	Year	Citation
**Mouse**	HFD	Liraglutide	↓ Hepatic TG, improved steatosis, ↓ inflammation	2014–2015	[103,104,105]
	HFD	Exenatide analog:AC3174	Ameliorated hepatic endpoints in NASH models	2012	[86]
	HFD + Fructose + Cholesterol	Tirzepatide	↓ Body/liver weight, ↓ hepatic lipids, ↓ glucose	2025	[106]
	ApoE KO + HFD	Liraglutide	Prevented MASLD, improved insulin sensitivity	2019	[107]
	STZ + HFD	Liraglutide	Improved steatosis histology, ↓ hepatic TG and cholesterol	2024	[108]
**Rat**	DIO	Semaglutide + PYY3-36	↓ Weight, steatosis, inflammation, improved IR	2025	[109]
	DIO	Liraglutide	Prevented MASLD, ↓ liver fat	2020	[110]
	HFD	Exenatide	Improved liver health, reduced steatosis	2014	[111]
**Zebrafish**	HFD, high-fat + cholesterol diet	-	Reduced hepatic lipid accumulation	2023–2010–2015	[112,113,114]
**Rabbit**	High-Fat, HCD	Exenatide	Reduced liver fat, improved metabolic parameters	2023–2010–2006	[115,116,117]
**Pig**	Leptin-deficient metabolic model	-	Attenuated MASH, improved insulin sensitivity	2023	[118]

High-Fat Diet: HFD; STZ: Streptozotocin; DIO: Diet-Induced Obesity; HCD: High-Cholesterol Diet; OA: Oleic Acid; GLP-1Ras: GLP-1 Receptor Agonists. The downward arrow (↓) indicates a decrease.

**Table 3:** Key Clinical Trials of GLP-1 Receptor Agonists (GLP-1RAs) in MASLD.

Trial/Study	Population	Study Design & Duration	Intervention	Primary Endpoint	Key Findings	Citation/Year
**LEAN** (Liraglutide Efficacy and Action in NASH)	52 overweight patients with biopsy-proven NASH	Double-blind RCT, 48 weeks	Liraglutide 1.8 mg QD s.c. vs. placebo	Biopsy-proven resolution of NASH without worsening fibrosis	NASH resolution in 39% vs. 9% (placebo); fibrosis progression lower with liraglutide (9% vs. 36%)	[119]-2016
**Lira-MASLD**	68 patients with T2D and MASLD	RCT, 6 months	Liraglutide 1.2 mg QD vs. intensified insulin	Change in hepatic fat content (H-MRS)	31% reduction in HFC with liraglutide; no change with insulin; HFC reduction correlated with weight loss	[120]-2016
**D-LIFT**	64 patients with T2D and MASLD	Open-label RCT, 24 weeks	Dulaglutide 0.75 mg (1.5 mg QW + standard care vs. standard care)	Change in HFC	Absolute HFC −3.5%; relative HFC456- −26.4%; significant reduction in GGT	[121]-2020
**Guo et al.**	128 patients with MASLD and uncontrolled T2D on metformin	RCT, 26 weeks	Liraglutide 1.8 mg vs. insulin glargine vs. placebo	Change in HFC (H-MRS)	Significant HFC reduction with liraglutide; improved ALT, AST, and HOMA-IR	[122]-2020
**AWARD (post hoc analysis)**	1499 T2D patients with MASLD/NASH	Post hoc analysis of AWARD-1, -5, -8, -9; 6 months	Dulaglutide 1.5 mg QW vs. placebo	Change in liver enzymes (ALT, AST, GGT)	Significant reductions in ALT, AST, and GGT vs. placebo	[123]-2018
**Efinopegdutide vs. Semaglutide**	145 patients with MASLD (HFC ≥ 10%)	Open-label, active-controlled RCT, 24 weeks	Efinopegdutide 10 mg QW vs. Semaglutide 1 mg QW	Relative reduction in HFC	HFC reduction: 72.7% vs. 42.3% (*p* < 0.001); similar weight loss	[124]-2023
**SURPASS-3 MRI**	T2D + MASLD (*n* = 296)	A randomized, open-label, active-controlled phase 3 sub-study of SURPASS-3, with a treatment duration of 52 weeks.	Tirzepatide	Reduction of the liver fat by 8.09% vs. 3.38%	Tirzepatide significantly reduced liver fat content (MRI-PDFF) and improved metabolic parameters compared with insulin degludec in patients with T2D.	[126]-2022

MASLD, metabolic dysfunction–associated steatotic liver disease; MASH, metabolic alcoholic steatohepatitis; T2D, type 2 diabetes; QD: once daily; QW: once weekly; HFC, hepatic fat content; H-MR: proton magnetic resonance spectroscopy; GLP-1Ras: GLP-1 Receptor Agonists.

## Data Availability

The original contributions presented in this study are included in the article. Further inquiries can be directed to the corresponding author.

## Abbreviations

ACC	Acetyl-CoA Carboxylase
ACTF4	Activating Transcription Factor 4
ALT	Alanine Aminotransferase
AML12	Alpha Mouse Liver 12 Cell Line
AMPK	AMP-Activated Protein Kinase
AST	Aspartate Aminotransferase
AWARD	Assessment of Weekly Administration of LY2189265 (Dulaglutide) in Diabetes
β-cells	Pancreatic Beta Cells
BMI	Body Mass Index
cAMP	Cyclic Adenosine Monophosphate
CHOP	C/EBP Homologous Protein
CREB	cAMP Response Element-Binding Protein
CVD	Cardiovascular Disease
DGAT1/2	Diacylglycerol Acyltransferase 1/2
D-LIFT	Dulaglutide Effect on Liver Fat in Type 2 Diabetes
DPP-4	Dipeptidyl Peptidase-4
EA	Elaidic Acid
ER	Endoplasmic Reticulum
Ex-ER	Exenatide Extended-Release
FFA	Free Fatty Acid
FDA	Food and Drug Administration
FOXA1	Forkhead Box A1
FOXO1	Forkhead Box O1
FAS	Fatty Acid Synthase
G6Pase	Glucose-6-Phosphatase
GGT	Gamma-Glutamyl Transferase
GIP	Glucose-Dependent Insulinotropic Polypeptide
GLP-1	Glucagon-Like Peptide-1
GLP-1R	Glucagon-Like Peptide-1 Receptor
GLP-1RA	Glucagon-Like Peptide-1 Receptor Agonist
GRP78	Glucose-Regulated Protein 78
HCC	Hepatocellular Carcinoma
HepG2	Human Hepatocellular Carcinoma Cell Line
HFC	Hepatic Fat Content
HNF4α	Hepatocyte Nuclear Factor 4 Alpha
HO-1	Heme Oxygenase-1
HOMA-IR	Homeostasis Model Assessment of Insulin Resistance
Huh7	Human Hepatoma Cell Line
IgG4	Immunoglobulin G4
IR	Insulin Resistance
IRS	Insulin Receptor Substrate
JS-1	Hepatic Stellate Cell Line
L02	Normal Human Liver Cell Line
LPS	Lipopolysaccharide
MASLD	Metabolic Dysfunction–Associated Steatotic Liver Disease
MASH	Metabolic Dysfunction–Associated Steatohepatitis
MET	Metformin
MRI-PDFF	Magnetic Resonance Imaging–Proton Density Fat Fraction
MS	Metabolic Syndrome
NQO1	NADH Quinone Dehydrogenase 1
Nrf2	Nuclear Factor Erythroid 2–Related Factor 2
OA	Oleic Acid
PA	Palmitic Acid
PEPCK	Phosphoenolpyruvate Carboxykinase
PHHs	Primary Human Hepatocytes
PKA	Protein Kinase A
PPARα/PPARγ	Peroxisome Proliferator-Activated Receptor Alpha/Gamma
QW	Once Weekly
QD	Once Daily
RCT	Randomized Controlled Trial
ROS	Reactive Oxygen Species
SCD1	Stearoyl-CoA Desaturase-1
SHP	Small Heterodimer Partner
SREBP-1c	Sterol Regulatory Element-Binding Protein-1c
T2D	Type 2 Diabetes
TCF4	T-Cell Factor 4
